# The rosetta stone of successful ageing: does oral health have a role?

**DOI:** 10.1007/s10522-023-10047-w

**Published:** 2023-07-08

**Authors:** Maximilian Poser, Katie E. A. Sing, Thomas Ebert, Dirk Ziebolz, Gerhard Schmalz

**Affiliations:** 1 Department of Cariology, Endodontology and Periodontology, University Leipzig, Liebigstr. 12, 04103 Leipzig, Germany; 2 Department of Medicine, Royal Devon and Exeter Hospital, University of Exeter Medical School, Exeter, EX2 5DW UK; 3 Medical Department III – Endocrinology, Nephrology, Rheumatology, University of Leipzig, Liebigstr. 20, 04103 Leipzig, Germany

**Keywords:** Ageing, Centenarians, Successful ageing, Inflammation, Oral health, Hallmarks of ageing

## Abstract

Ageing is an inevitable aspect of life and thus successful ageing is an important focus of recent scientific efforts. The biological process of ageing is mediated through the interaction of genes with environmental factors, increasing the body’s susceptibility to insults. Elucidating this process will increase our ability to prevent and treat age-related disease and consequently extend life expectancy. Notably, centenarians offer a unique perspective on the phenomenon of ageing. Current research highlights several age-associated alterations on the genetic, epigenetic and proteomic level. Consequently, nutrient sensing and mitochondrial function are altered, resulting in inflammation and exhaustion of regenerative ability.

Oral health, an important contributor to overall health, remains underexplored in the context of extreme longevity. Good masticatory function ensures sufficient nutrient uptake, reducing morbidity and mortality in old age. The relationship between periodontal disease and systemic inflammatory pathologies is well established. Diabetes, rheumatoid arthritis and cardiovascular disease are among the most significant disease burdens influenced by inflammatory oral health conditions. Evidence suggests that the interaction is bi-directional, impacting progression, severity and mortality. Current models of ageing and longevity neglect an important factor in overall health and well-being, a gap that this review intends to illustrate and inspire avenues for future research.

## Background

The attainment of very old age and accumulation of life experiences has always been fascinating to humankind. Throughout history humans have revered longevity. Elevated status of elders and ancestors in the late neolithic period or the pronounced role in culture (Xiao 2023), religion (James and Version 2004) or society (P C 2003) illustrate that point.

Research continues to examine the question: How do people achieve exceptional longevity and age successfully? Although many questions remain to be answered, metabolic dysregulation (Paolisso et al. 2000), inflammation (Arai et al. 2015; Franceschi et al. 2007), altered immune function (Salvioli et al. 2013; Wikby et al. 2005), endocrine changes (Paolisso et al. 1997; Suzuki et al. 2001) and increased oxidative stress (Belenguer-Varea et al. 2020) all seem to play a significant role in ageing. Social relationships (Holt-Lunstad et al. 2010), genetic background (Terry et al. 2004), posttranslational modifications (Ben-Avraham et al. 2017) and the interaction between our genes and the environment(Ebert et al. 2022; Christensen et al. 2006; Herskind et al. 1996a) also appear to have a large impact. Previous research has partially uncovered some influential factors, however the entire complexity of attaining extreme longevity remains elusive.

Furthermore, the factors that influence extreme longevity have received different amounts of attention, with some influential factors having been omitted. One of the neglected areas could be the importance of oral health in achieving very old age. The following narrative review will illustrate the current research in the oldest old and discuss a missing piece of the puzzle, that might help to gain a more comprehensive and deeper understanding.

## How to characterize successful ageing?

Ageing is a complex process, in order to discover causative factors, a clear definition of “successful ageing” is vital. This would need to encompass the various pathways leading to exceptionally old age. In the current literature a multitude of different definitions exist, depending on the cultural, social or biological context of the examined hypothesis (Herskind et al. 1996b; Estebsari et al. 2020).

A recent review by Pignolo et. al. proposed two overarching criteria for attaining exceptional longevity: (1) biological age < chronological age, (2) slowed or delayed decline in functional status (López-Otín and Kroemer 2021). Considering the work of Rowe and Kahn, the functional aspect can be further divided into 3 subcategories: minimize risk of disease and disability, maintain physical and cognitive function and continuous engagement in life (Pignolo 2019). These assumptions seem to be the best approximation, considering they are common clinical features in centenarians. They offer potential endpoints for further research. For example, molecular tests like methylation clocks could provide additional insight and a more precise definition, as they already have in the definition of biological age (Rowe and Kahn 1997).

The ability to sustain health for longer, appears to be a core feature of exceptionally long-lived individuals. Supporting this idea, Fries et al. described a compression of morbidity, hypothesizing that the onset of chronic morbidity at a later stage in life, is greater than their gain in life expectancy, resulting in a smaller fraction of life spent with illness (Gutman et al. 2020). Recent evidence in a cohort aged 65 and over showed a reduction in disability by 2% a year (Fries 1980), and a reduced need for help with activities of daily living (ADLs), compared to the same age cohort 10 years prior (Fries 2005; Schoeni et al. 2001; Waidmann and Liu 2000; Cutler 2001; Freedman et al. 2006). These results are based on data between 1982 and 2005 (Fries 2005; Schoeni et al. 2001; Waidmann and Liu 2000; Cutler 2001; Freedman et al. 2006) and supports the notion of compressed. Further evidence suggests a slowing of this trend in recent years (Schoeni et al. 2008; Cai and Lubitz 2007; Fuller-Thomson et al. 2009).

The compression of morbidity is accentuated in increased age and therefore greatest in centenarians (Martin and Schoeni 2010), although the underlying mechanisms may differ (Andersen et al. 2012).

## Lessons learned from centenarians

Due to the perceived significance of reaching 100 years, there are many non-verifiable claims of individuals reaching exceptional lifespans. The most reliable reports suggest that the oldest recorded individuals were *Jeanne Calment* as the longest living women, at 122 years and 165 days (Terry et al. 2008) and *Jiroemon Kimura* as the longest living man, at 116 years and 54 days (Robine and Allard 1998). Previously it has been estimated, that the likelihood of reaching 100 years of age for people born at the turn of the last century in North America was 7 in 1000 (0,7%). For birth cohorts born at the turn of the current millennia, it has been projected that 1 in 2 will reach the remarkable age of 100 (Gondo et al. 2017). With the increasing number of centenarians, it can be expected that the prevalence of the very rare supercentenarians (age > 110 years), currently estimated at 1 in 5 million, will increase as well (Christensen et al. 2009).

One of the major differences between centenarians and the rest of the ageing population is a pronounced compression of morbidity. Andersen et al. were able to illustrate this phenomenon in centenarians (> 100), semi-super centenarians (> 105) and supercentenarians (> 110), showing that the greatest delay in the onset of disease appeared in the oldest sub-cohorts. In this study, researchers postulated that supercentenarians sustain a health span that approximates their life span (Martin and Schoeni 2010).

People who have attained exceptional longevity may be classified by different morbidity profiles. Evert et al. described these profiles as: survivors, delayers and escapers. The cohort of survivors were those who had a diagnosis of an age-related disease before the age of 80 and survived into their hundreds. Delayers were diagnosed with an age-related disease between the ages of 80 and 100 and escapers weren´t diagnosed with an age-associated illness until they were over 100 years old (Young et al. 2010). It appears that the percentage of escapers rises with the age of the studied cohort. While 50% of centenarians in the New England Centenarian Study (NECS) were described as delayers, approximately 70% of the supercentenarians fitted the criteria for escapers (Martin and Schoeni 2010). This shows that there might be distinct differences in survival mechanisms within the oldest old.

In contrast, there are remarkable similarities between centenarians. A clustering of centenarians in certain areas of the world may be observed, these are referred to as blue zones (Evert et al. 2003).

Whilst the habitants in a blue zone may share a common genetic background, they also exhibit similarities in behavioral patterns that are considered to further longevity. These include: eating in moderation, mostly plants, exercise as part of a daily routine, purposeful living, maintaining a supportive social circle, spirituality and keeping a healthy BMI throughout their lifetime (López-Otín and Kroemer 2021; Evert et al. 2003). The overlap between nutrition and oral health is especially interesting and research has focused on analyzing the diet of the oldest old. The diet of a well renowned cluster of centenarians in Okinawa (Japan) is characterized by low energy density and is nutrient-rich, with a high content of antioxidants consisting mainly of sweet potatoes, vegetables and legumes. Additionally, estimations that this cohort managed to live with an approximately 11% caloric restriction over their lifetime exist, helping to maintain a low BMI and potentially furthering longevity (Buettner and Skemp 2016; Willcox et al. 2007; Willcox et al. 2014; Willcox et al. 2009; Willcox et al. 2017; Willcox and Willcox 2014). In contrast, recent lifestyle changes reversed this trend in younger generations, leading to increased appearance of diabetes and obesity (Willcox et al. 2009; Longo and Anderson 2022).

## General hypotheses for successful ageing

As described previously, ageing is a dynamic process with a multitude of influential factors. One of the most important modulators of ageing appears to be inflammation. Arai et al. showed that low-level inflammation is the best predictor of all-cause mortality, capability and cognition in the very old and (semi-) supercentenarians (Arai et al. 2015). This landmark study confirmed the findings from younger and smaller cohorts (Willcox et al. 2012; Jenny et al. 2012; Akbaraly et al. 2013; Schnabel et al. 2013), illustrating the central role of inflammation in the ageing process. Interestingly, they managed to show that centenarians, who escaped morbidity and mortality longer, show higher levels of systemic inflammation markers. The authors theorize that whilst centenarians may be able to avoid low-grade inflammation for the majority of their life, they may still exhibit a rise in proinflammatory processes towards the end of their lifespan similar to the general population (Arai et al. 2015). Other hypotheses postulate that high levels of anti-inflammatory molecules might counteract the detrimental effect of high systemic inflammation in centenarians, therefore negating the mortality risk (Salvioli et al. 2013).

Another factor contributing to the rise of inflammatory markers in advanced age seems to be a dysregulation of the immune system and immunosenescence. In addition to the increased percentage of late T effector cells, memory T cells (Varadhan et al. 2014) myeloid cells and NK cells (Franceschi et al. 2000), the decrease in naïve lymphocytes (Varadhan et al. 2014) and B cells (Franceschi et al. 2000) can largely explain the reduced response to new antigens in old people (Salvioli et al. 2013). This results in an overall increased mortality risk (Wikby et al. 2005). Large cohort studies of elderly people in Scandinavia described an Immune Risk Phenotype (IRP), constituting an inversion of CD4/CD8 ratio, poor T cell proliferation response and persistent CMV infection, associated with increased risk of mortality (Wikby et al. 2005). Centenarians appear to be free of the IRP, implicating an impact of immundysregulation on the process of ageing (Larbi et al. 2008).

As already noted, maintaining a normal BMI seems to be a common denominator in the oldest old (Paolisso et al. 2000; Strindhall et al. 2013). Adipose tissue is known to contribute to the inflammatory load, partly due to an infiltration by macrophages, but also as a result of a metabolic reaction to food abundance (Paolisso et al. 1995). Hallmarks of this metabolically induced inflammation are: (1) orchestration by metabolic cells in response to excess energy and nutrients, (2) low grade inflammation, (3) chronic, proinflammatory milieu (Paolisso et al. 1995). This ‘metaflammation’ correlates with the onset of metabolic syndrome and Type 2 Diabetes (T2DM) (Gregor and Hotamisligil 2011). In contrast, insulin mediated glucose uptake appears to be preserved in healthy centenarians (Hotamisligil 2006), illustrating the importance of maintaining a healthy weight and body composition into old age. However, according to the obesity paradox, moderately elevated BMI is associated with a lower mortality in certain disease states e.g., cancer, end stage kidney disease and cardiovascular disease (Paolisso et al. 1996; Lennon et al. 2016; Park et al. 2014). This idea has been criticized because BMI is a relatively crude measure and fails to sufficiently incorporate the influence of body composition and metabolic health (Paolisso et al. 1996; Lavie et al. 2016; Bosello and Vanzo 2021; Elagizi et al. 2018; Caan et al. 2018).

Globally women have a far greater probability of surviving into old age (Bowman et al. 2017; Austad and Bartke 2015), although female centenarians are on average less healthier than their counterparts (Martin and Schoeni 2010; Austad and Fischer 2016). It is thought that women have the ability to deal more successfully with age-related morbidity and functional decline, that often leads to mortality in men at a younger age (Franceschi et al. 2000; Perls 1995). Additionally, it has been postulated that the relatively smaller build of a woman results in a lower GH secretion, which is positively associated with longevity (Perls and Fretts 1998; Bartke 2008; Bartke et al. 2013). Studies from nematodes (*Caenorhabditis elegans*), flies (*Drosophila melanogaster*) and mice (*Mus musculus*) demonstrate that a disruption of the IGF1 signaling pathway results in an increased lifespan among several species (Spoel et al. 2016; Kimura et al. 1997; Kenyon 2001). This further supports the key role of the GH-IGF1 axis in ageing. Studies of patients with Laron Syndrome (congenital IGF 1 deficiency) suggest a high degree of protection against cancer, T2DM and pro-ageing signaling (Clancy et al. 2001).

There are several genes implicated in the attainment of extreme longevity, however twin studies attribute only 25% of lifespan variance to genetic factors (Ebert et al. 2022; Christensen et al. 2006; Herskind et al. 1996a). It therefore follows that a much larger proportion must be derived through other mechanisms. In a landmark paper from López-Otín et al. from 2013, nine hallmarks of ageing have been described. These hallmarks mediate the influence that the environment has on the ageing process within the cell (Guevara-Aguirre et al. 2011).

Whilst these hallmarks partly explain the ageing process by predicting the cumulative effect of age related morbidity (López-Otín et al. 2013), other researchers have described them as insufficient (Fraser et al. 2022). At the Copenhagen ageing meeting in March 2022 these criticisms were addressed through expansion of the original hallmarks and the addition of 5 further hallmarks (Gems and Magalhães 2021).

## Hallmarks of ageing

The nine original hallmarks of ageing are genomic instability, telomere attrition, epigenetic alteration, loss of proteostasis, deregulated nutrient-sensing, mitochondrial dysfunction, cellular senescence, stem cell exhaustion and altered intercellular communication (Guevara-Aguirre et al. 2011). The recently added hallmarks include compromised autophagy, dysregulation of RNA splicing, microbiome disturbance, altered mechanical properties and inflammation (Gems and Magalhães 2021). The underlying assumption is that the general cause of age-related phenomena is the accretion of cellular damage in a time dependent manner (Schmauck-Medina et al. 2022; Gems and Partridge 2013; Kirkwood 2005). The hallmarks can be described as different manifestations of this lifelong attrition.

Genomic instability results from the cumulative insults to the genetic material (Vijg and Campisi 2008). It can be caused by exogenous factor e.g. physical agents or endogenous processes e.g. DNA replication errors (Moskalev et al. 2012). The damage can effect nuclear DNA, mitochondrial DNA or the integrity of the nuclear architecture itself and thereby causing age-associated pathologies (Guevara-Aguirre et al. 2011).

The notion of instability also relates to cell architecture, altering the mechanical properties of cells and tissues. Impairments of the cytoskeleton disrupt cell motility and intercellular communication (Gems and Magalhães 2021). Similarly, the age-related alterations of the extracellular matrix caused for example by glycation cross-links, have been shown to substantially alter cell behavior (Hoeijmakers 2009) and are linked to age-associated diseases (Jain 2018).

Telomeres are thought to be especially susceptible to age-related impairment. Damage present in these regions is notoriously hard to repair due to the presence of shelterins, preventing access of repair enzymes (Bahour et al. 2022). Additionally, the length of telomeres has been found to shorten with advancing age, imposing a proliferative limit (Fumagalli et al. 2012), influencing the regenerative ability of tissues (Hayflick and Moorhead 1961) and promoting senescence (Bahour et al. 2022).

Epigenetic alterations are associated with ageing (Armanios and Blackburn 2013; Fraga and Esteller 2007) Histone modification and the resulting expression of certain genes has been linked to prolonged lifespan. Sirtuins have become one of the major focus of research in this area, influencing for example genomic stability or glucose metabolism (Han and Brunet 2012; Kanfi et al. 2012, 2010). Furthermore, alteration in DNA methylation, chromatin remodeling and transcriptional alterations have been identified as targets for ageing research (Guevara-Aguirre et al. 2011).

An additional layer of dysregulation arises in RNA processing, (Zhong et al. 2010) further disrupting the control of gene expression. Alterations of mRNA influence the ageing process (Gems and Magalhães 2021) and have been implicated in cancer growth (Holly 2013) and cell senescence (Yuan et al. 2019; Shen et al. 2019).

The loss of proteostasis also appears to be a feature of ageing (Latorre et al. 2017; Powers et al. 2009). Key systems for protein stability, the chaperone system and disposal, the autophagy-lysosomal system and ubiquitin–proteasome system display a reducing activity with advancing age (Koga et al. 2011; Calderwood et al. 2009; Tomaru et al. 2012). Their reactivation has been identified as a potential target for pharmacological intervention (Rubinsztein et al. 2011).

In a recent publication by Schmauck-Medina et al. compromised autophagy is considered a hallmark in its own right (Gems and Magalhães 2021). It acts as a central component of several age-related conditions for example immunosenescence and neurodegenerative disease (Johnson et al. 2015; Wong et al. 2020). Autophagy stands at the intersection between hallmarks, influencing the regulation of a selection of cellular functions, including DNA repair, cellular stress-response and glucose and lipid metabolism (Aman et al. 2021).

Deregulated nutrient-sensing manifests itself in the aforementioned Insulin and IGF1 signaling system, which plays a role in glucose sensing, as well as other important systems that mediate the ageing process. Among these the mTOR pathway is responsible for sensing high amino acid concentrations, indicative of a fed state. In contrast, the AMPK pathway is activated by high AMP levels and sirtuins by high NAD^+^ levels, both of which are surrogate parameters for low energy states (Kaushik 2018). These are some of the most promising targets for research with possible dietary (Houtkooper et al. 2010; Fontana et al. 2010; Weindruch and Walford 1982; Colman et al. 2009) and pharmacological interventions (Rubinsztein et al. 2011; Yin and Klionsky 2022; Johnson et al. 2013).

Mitochondrial dysfunction appears to be a characteristic of ageing, implicating diminishing ATP production and electron leakage, due to increasing inefficiency of the respiratory chain (Walters et al. 2016). This potentially increases reactive oxygen species (ROS) to detrimental levels (Green et al. 2011) and defective mitochondrial biogenesis (Hekimi et al. 2011; Wang and Klionsky 2011). Additionally, mitochondrial homeostasis seems to be intimately intertwined with the nutrient sensing system through SIRT1 (Sahin and DePinho 2012; Rodgers et al. 2005), SIRT3 (Lee et al. 2008; Lombard et al. 2007; Giralt and Villarroya 2012) and AMPK (Qiu et al. 2010). Altered mitochondrial function represents a vital intersection between several mechanisms of ageing and may further stem cell depletion (Hawley et al. 2010; Fang et al. 2014; Scheibye-Knudsen et al. 2015).

Cellular senescence is the permanent arrest of the cell cycle with stereotypic phenotypic alterations (Lou et al. 2020; Campisi 2007; Collado et al. 2007). Telomere shortening, DNA damage outside the telomere region and increased mitogenic signaling due to for example the de-repression of the INK4/ARF locus are known to be a causative factor of cellular senescence (Campisi 2007; Kuilman et al. 2010). Senescence can be seen as a compensatory mechanism to arrest damaged cells and replace them. With increasing age, the balance of arrest and replacement is disturbed, leading to accumulated senescent cells. Due to their prolific pro-inflammatory secretory ability, they may contribute to further ageing. This phenomenon has been described as the “senescence-associated secretory phenotype” (Collado et al. 2007; Gorgoulis and Halazonetis 2010).

Stem cell exhaustion leading to reduced regenerative ability of tissue, appears to be another characteristic of the ageing process. It is thought to be the consequence of the accumulation of different insults e.g. DNA damage (Rodier and Campisi 2011) over a lifetime, (Guevara-Aguirre et al. 2011) leading to a reduced proliferation (Rodier and Campisi 2011) or inadequate hyperproliferation further depleting the stem cell reserve (Rossi et al. 2007).

Altered intercellular communication is an integrative part of ageing. Alterations appear on the neuronal, neuroendocrine and endocrine level (Rera et al. 2011; Zhang et al. 2013; Russell and Kahn 2007; Rando and Chang 2012). One of the major manifestations and drivers is inflammation (Laplante and Sabatini 2012). Other forms of cell–cell communications are known to further drive the ageing process e.g. over gap junction mediated cell–cell contact with ROS (Salminen et al. 2012) and exosome regulated communication (Nelson et al. 2012; Duggan et al. 2022; Xu and Tahara 2013). Altering intercellular communications will be an important target for potential interventions (Wang et al. 2021a; Piper et al. 2011; Sanchez-Roman et al. 2012; Loffredo et al. 2013).

In the 2022 publication by Schmauck-Medina et al. inflammation is considered a separate hallmark (Gems and Magalhães 2021). High levels of proinflammatory markers like IL-1, IL-6, C-reactive protein correlate with ageing (Arai et al. 2015; Claesson et al. 2012). Furthermore, age-dependent chronic inflammation, also known as inflammaging, has been implicated in a wide range of diseases (Franceschi et al. 2007, 2017; Laplante and Sabatini 2012; Ferrucci and Fabbri 2018; Leonardi et al. 2018).

Microbiome disturbances are thought to be associated with inflammation, this is more pronounced with age-associated structural impairment of barriers e.g. blood brain barrier (Gems and Magalhães 2021; Teissier et al. 2022). Recent advances in sequencing technology resulted in the discovery of age related changes in the gut microbiome, notably a shift of microbial populations and a reduction of diversity (Zhu et al. 2020).

## Oral conditions in medicine

According to the FDI World Dental Federation: ‘Oral health is multifaceted and includes the ability to speak, smile, smell, taste, touch, chew, swallow, and convey a range of emotions through facial expressions with confidence and without pain, discomfort, and disease of the craniofacial complex.’ (Wilmanski et al. 2021) A range of disease states impact oral health, most notably dental caries, tooth loss, periodontal disease, oral and dental trauma, oral cancer, noma and birth defects (Glick et al. 2017). Oral health has been recognized as playing a substantial role in overall health, wellbeing and quality of life (Wilmanski et al. 2021; Health and [https:, , www.who.int, health-topics, oral-health#tab=tab_1; Petersen and Kwan 2009). In 2019 the Global Burden of Disease was estimated at 3.5 billion affected individuals for oral disease, one of the most common non-communicable diseases (NCDs) worldwide (Peres et al. 2019). Unfortunately, medical training often neglects oral and dental medicine. This leaves doctors with very little knowledge regarding an important influencing factor of their patients´ general health (Dörfer et al. 2017; GBD 2019).

In contrast, dentistry is moving away from a disease-based treatment model, towards individualized prevention, beginning to emphasize the systemic implications of oral disease (Doshi et al. 2019; Ahluwalia et al. 2016).

**Fig. 1:**
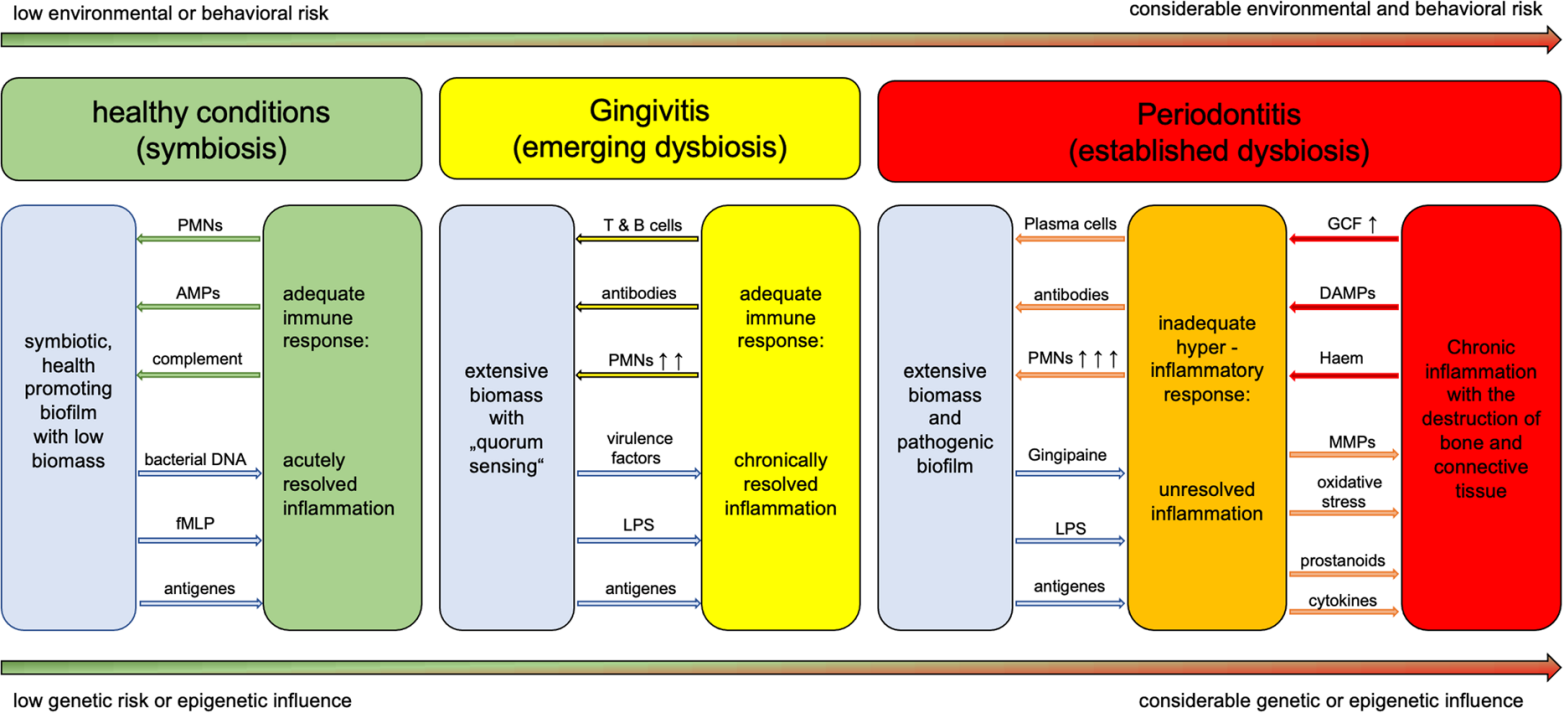
**Current model for host bacteria interaction in the pathogenesis of Periodontitis**. Extensive biomass drives a proportionate immune response resulting in gingivitis. Without the removal of the biofilm an increase in pathogenicity occurs, resulting in tissue destruction and periodontitis. *PMNs* polymorphonuclear neutrophils, *AMPs* antimicrobial peptides, *fMLP* N-formylmethionyl-leucyl-phenylalanin, *LPS* lipopolysaccharide, *GCF* gingival crevicular fluid, *DAMPs* damage-associated molecular patterns, *MMPs* matrix metalloproteinases (Schmalz and Ziebolz 2020)

**Fig. 2:**
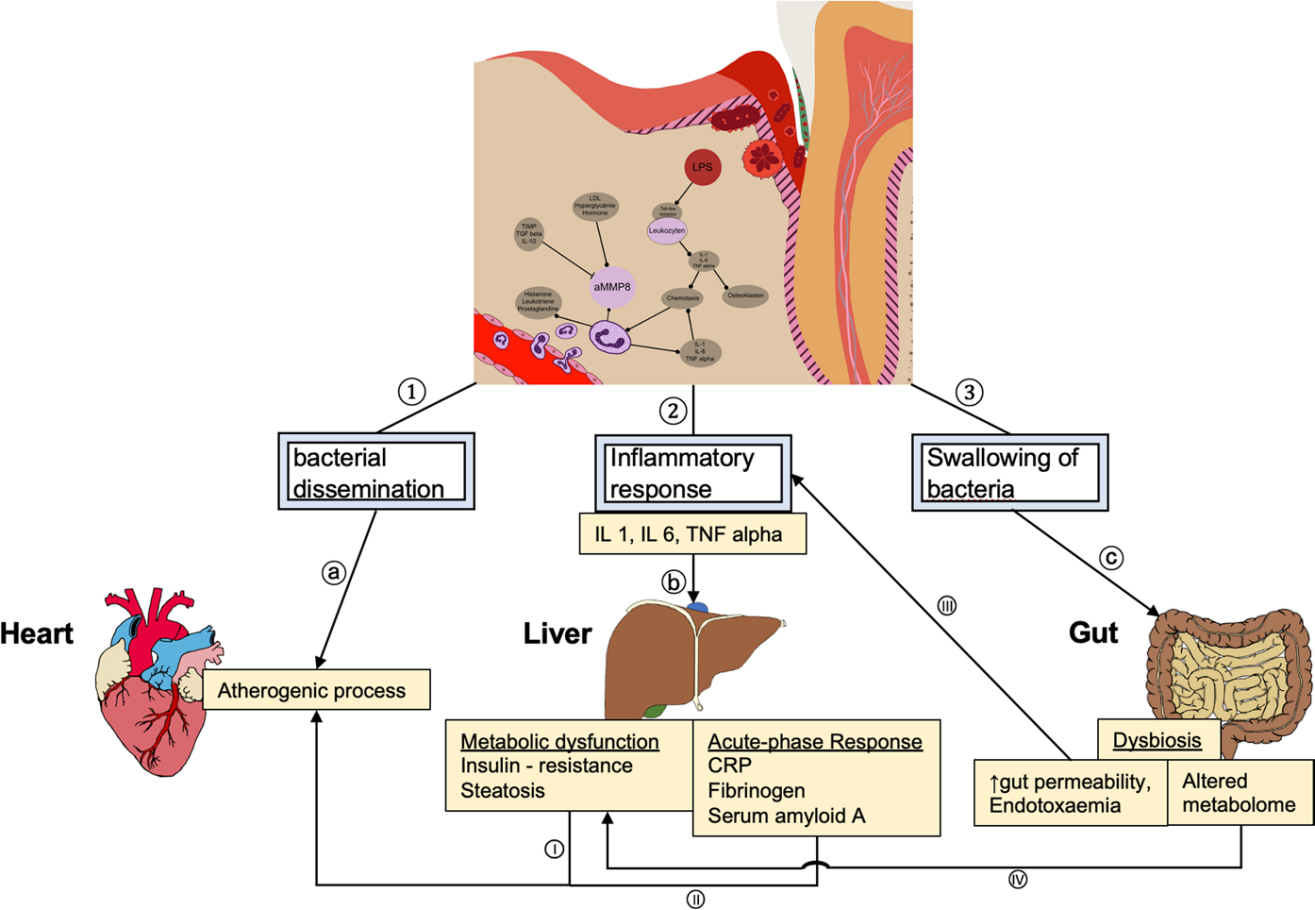
**Periodontitis and systemic implications**. Periodontitis occurs as a consequence of the imbalance between the subgingival microbiota and the host immune response (Gondo et al. 2006; Arnold et al. 2010; Hajishengallis 2021). The commensal bacteria population shifts into a pathogenic dysbiotic state (Dyke et al. 2020), stimulating an inflammatory response. This results in a local accumulation of leucocytes, specifically neutrophiles (Gondo et al. 2006). They contribute to the tissue destruction through the secretion of Matrix-Metalloproteinases (MMPs) and reactive oxygen species (ROS) (Lamont et al. 2018; Curtis et al. 2000). The failure to control the dysbiotic environment leads to tissue penetration of bacteria. Through the interaction with macrophages and dendritic cells an increased production of cytokines, e.g. Interleukin 1 (IL1), Interleukin 6 (IL6) and tumor necrosis factor alpha (TNF ⍺) occurs. Furthermore, proinflammatory cytokines promote chemotaxis, increasing immune cell recruitment and aggravating the inflammatory process (Curtis et al. 2000; Hajishengallis and Hajishengallis 2014). **1)** In periodontitis the risk of bacteraemia and systematic dissemination of bacteria is increased (Hajishengallis 2014). **a** In addition, further dissemination occurs through survival in phagocytic host cells. Consequently, periodontal bacteria can be found in atherosclerotic lesions, possibly aggravating the process (Pelletier et al. 2010). **2)** The local inflammatory response consisting of IL 1, IL6 and TNF ⍺ can enter the circulation and possibly induces changes in the **b** liver. Thus, leading to metabolic dysfunction expressed as insulin resistance (Gondo et al. 2006; Schenkein et al. 2000) and potentially triglyceride accumulation (Carrion 2012). This process aggravates atherogenesis. Additionally, circulation cytokines may induce an acute phase reaction in the liver, which in turn is marked by elevated levels of C reactive Protein, Fibrinogen and Serum Amyloid A, furthering the atherosclerotic processes. **3)** The swallowing of large quantities of bacteria present in periodontitis, is known to influence the gut microbiome, **c** leading to dysbiosis (Gondo et al. 2006; Jepsen et al. 2000). Altered gut microbiota has been linked to increased gut permeability and endotoxaemia due to an induced downregulation of tight junction proteins (Arimatsu et al. 2014; Yamazaki et al. 2021; Cani et al. 2008 Kashiwagi et al. 2021), further promoting a systemic inflammatory state (Jepsen et al. 2000). Dysbiosis has been related to changes in metabolites produced by the microbiota, which may increase insulin resistance independent of the effect of proinflammatory cytokines induced by the endotoxaemia (Arimatsu et al. 2014)

In addition, research regarding the relationship between periodontal disease and systemic conditions has moved into the focus of dental research, with links to as many as 57 systemic diseases currently under investigation (Schmalz et al. 2020). In general, periodontal disease can be described as a dynamic process leading to a dysbiosis resulting in chronic inflammation and destruction of the tooth supporting tissue (gingiva, periodontal ligament, alveolar bone) (Schmalz and Ziebolz 2020). (Fig. 1) During this process pathogens and inflammatory molecules may leak into the blood stream and potentially aggravate other present systemic diseases (Fig. 2). The bi-directional association between T2DM and periodontal disease is widely accepted (Loos 2016). In non-diabetic individuals the presence of periodontitis is associated with higher HbA1c and a higher risk of developing pre-diabetes and diabetes (Meyle and Chapple 2000). If diabetes is present, periodontitis is associated with poorer glycemic control and a higher risk of complications, including diabetic retinopathy, cardiovascular disease, ischemic stroke, neuropathic foot ulcers and chronic kidney disease (Lalla and Papapanou 2011; Graziani et al. 2018; Sanz et al. 2018, 2020; Borgnakke and Poudel 2021; Song et al. 2021; Dyke et al. 2021; Borgnakke et al. 2015; Nguyen et al. 2020; Zhao et al. 2020). Poor diabetic control is linked to accelerated attachment loss (Lalla and Papapanou 2011; Alvarenga et al. 2020). Obesity and metabolic syndrome have also been implicated with periodontal disease, possibly through the creation of a pro-inflammatory state (Genco and Borgnakke 2000; Demmer et al. 2012). The association between rheumatoid arthritis and periodontal disease is well documented (Arana et al. 2017; Dursun et al. 2016; Potempa et al. 2017). A common denominator is the presence of IL-1beta and tumor necrosis factor-alpha in both rheumatoid arthritis and periodontal disease (Potempa et al. 2017; Cheng et al. 2017), which also has been described as a feature of immunosenescence (Kaur et al. 2013; Mirrielees et al. 2010).

Proinflammatory states in patients with periodontitis have been associated with a stronger inflammatory response when challenged with bacteria or lipopolysaccharides (LPS) compared to healthy individuals (Fagiolo et al. 1993). Some research has suggested that this increased response could be the result of hyperreactive myeloid cells. This may be induced by epigenetic rewiring, following changes in cell metabolism, caused by bacteremia and systemic inflammation present in periodontitis (Zanni et al. 2003; Ling et al. 2015; Barutta et al. 2022; Netea et al. 2020). This process causes a prolonged hyperactivation of the innate immune response, recently described as “trained immunity” (Ling et al. 2015) and potentially increases inflammation and metabolic degradation present in periodontitis. Furthering immune cell activation and tissue destruction, reactive oxygen species (ROS) have been identified as playing a crucial role in periodontal inflammation (Saeed et al. 2014).

This offers a glimpse into the possible role of inflammation in periodontal disease and the ageing process as a potential point of interconnection.

With age being a major influence on oral conditions and tooth loss (Bekkering et al. 2018; Sczepanik et al. 2000; Kassebaum et al. 2014; Frazão et al. 2003; Barbato and Peres 2015; Silva et al. 2009), remarkably little research has been done in the cohort ageing most successfully.

## Oral conditions in centenarians: current state of knowledge

Oral medicine for the elderly encompasses a particular set of challenges. Influential factors include comorbidities, polypharmacy, age-associated cognitive impairment and a lower priority in an institutionalized setting (Haugejorden et al. 2003; Fure 2003; Schmalz et al. 2021). The edentulous rate in the elderly varies between 22 and 64% (Wong et al. 2019; Rantzow et al. 2018; Ziebolz et al. 2017; Fitzpatrick 2000; Hopcraft et al. 2012; Simunković et al. 2005; Rabiei and Kasemnezhad 2010) and is an important risk factor for malnutrition (Haugejorden et al. 2003; Wong et al. 2019; Northridge et al. 2012; Montal et al. 2006). Additionally, for those with remaining teeth, a high burden of periodontal disease and increased need for general dental treatment has been reported (Lamy et al. 1999; Cousson et al. 2012). Advancing age may also reduce the capacity to perform sufficient oral hygiene (Rantzow et al. 2018).

Very few publications on the oral health of centenarians exist. A systematic review from 2020 revealed only two papers fitting the search criteria (Jordan 2016). Since then, the same workgroup has published some of the first studies investigating the oral conditions of centenarians. They managed to show that a surprisingly large percentage of centenarians still had teeth (64%) with the mean number of teeth being 9.1 SD 7.1. Furthermore, most centenarians showed no or only moderate periodontal disease, with a minority of 19% having severe periodontitis (Micheelis 2011; Frese et al. 2020). Other recent studies in a different cohort found higher rates of edentulousness, suggesting that the cultural background may play a pivotal role in interpreting this data (Sekundo et al. 2020a). Although these findings are important, further research is needed to establish the complex interaction between systemic inflammation, nutrition and oral health.

Albani A et al. examined two studies from England (New Castle 85 + Study) and Japan (TOOTH) to illuminate the connection between oral and overall health. In the *Newcastle cohort* difficulty eating food due to dental problems, was associated with higher odds of frailty, mobility limitations, slower gait speed and weaker grip strength. For the *TOOTH cohort*: frailty, mobility limitations and slow gait speed showed a similar association. The risk of frailty was increased by two-thirds in the subcohort with no natural teeth remaining (Sekundo et al. 2020b). Although having a cross-sectional study design and distinct differences existing in the two studied cohorts e.g. edentulous rate, this publication still demonstrates an association between oral health and overall function (Sekundo et al. 2020b).

It must be noted that these studies were performed in people under 100 years and therefore the results may not necessarily apply to centenarians or (semi-)supercentenarians. More research is needed to understand the dental and periodontal health of the oldest people in our societies. Currently oral health and illness aren´t integrated into the complex construct of successful ageing.

## Synthesis of oral health and successful ageing

**Fig. 3:**
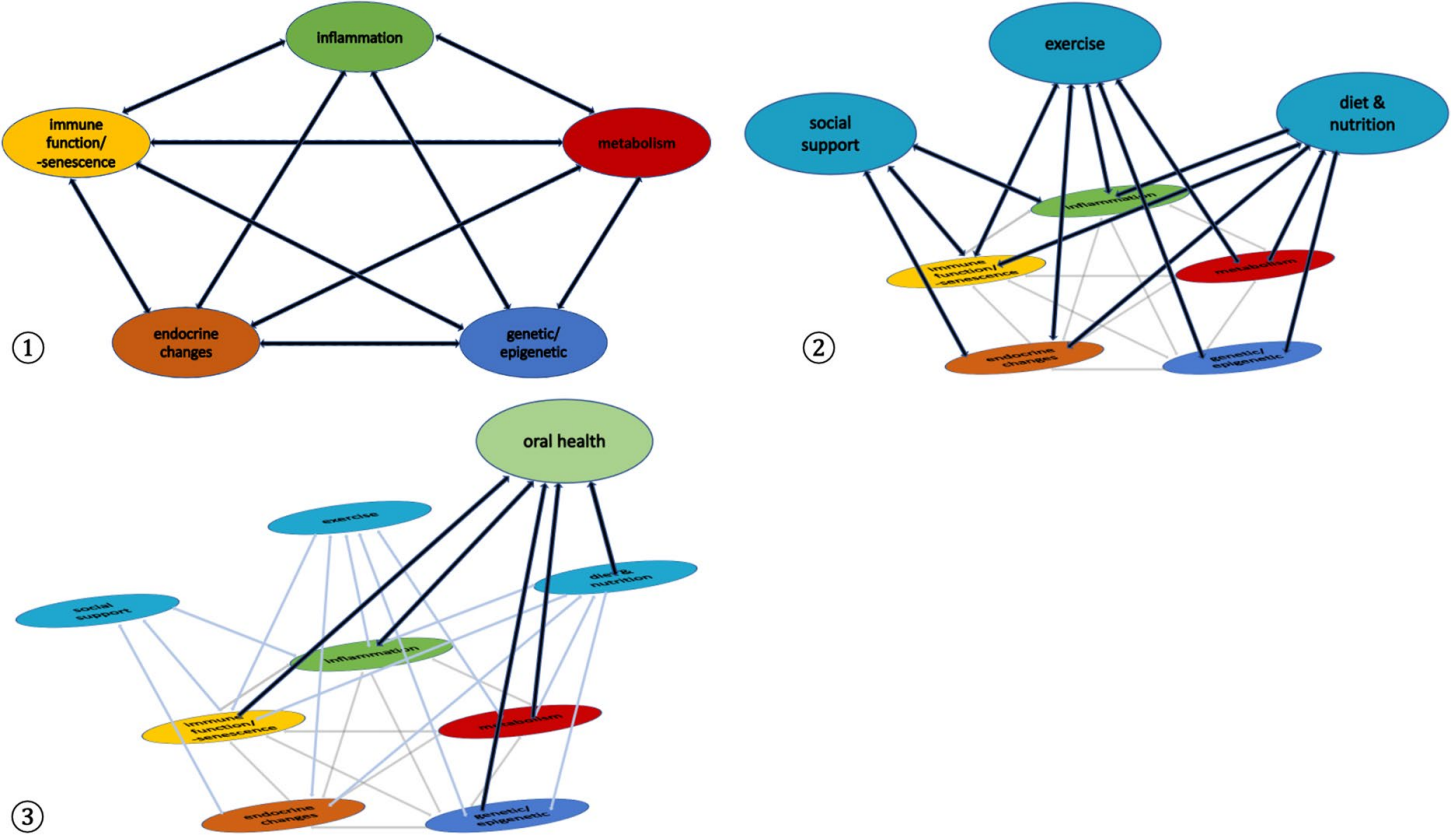
**Interconnections between ageing related processes and the potential role of oral health**. **1)** In ageing, multiple factors within a variety of systems are in a state of dynamic change. These links have been established between inflammation, immune function, endocrine changes, genetics/epigenetics and metabolic changes. **2)** Beneficial behavioral patterns e.g. frequent exercise have multiple positive effects. In the case of exercise; reduced inflammation, improved immune function, enhanced hormonal health, engaged epigenetic mechanisms improving memory function and better insulin sensitivity have been reported. Similar links exist for several other behavioral patterns e.g. social support, diet, purposeful living and spirituality. Oral health has an important role in preventing age related decline. **3)** Oral disease is associated with reduced masticatory function, leading to an impaired nutrient intake, especially protein. Periodontal disease increases the risk of T2DM and prediabetes in non-diabetics and has been linked to poorer glycemic control and a higher complication rate in diabetic patients. The presence of a proinflammatory state, characterized by interleukine-1beta and tumor necrosis factor-alpha, potentially influences autoimmune disease e.g. rheumatoid arthritis and immune senescence. Bacterial antigens and cytokines trigger changes in the cell metabolism of myeloid cells, leading to epigenetic changes, which ultimately enhance the inflammatory response. Recent research suggests that ageing is not only expressed on the macroscopic scale but also on the molecular level, providing possible targets for pro longevity interventions

To attain extreme longevity, maintenance of homeostasis appears to be a prerequisite. The ageing process can be approached as web of complex interactions between several factors (see Fig. 3). These might be attenuated by life-style choices, genetic predispositions and social support (see Fig. 3) The factors may either have a direct effect on the deterioration of the organism or influence ageing potentially through one or several processes described in the hallmarks of ageing. In the research by López-Otín et al. primary hallmarks include genomic instability, telomere attrition, epigenetic alterations and loss of proteostasis. They result from insults to the body leading to negative consequences. Antagonistic hallmarks can be seen as protective compensatory mechanisms responding to damage. These initially protective processes become detrimental to cellular function. Deregulated nutrient sensing, mitochondrial dysfunction, and cellular senescence are included in this category. Finally, altered intercellular communication and stem cell exhaustion are integrative hallmarks. They are the result of an accumulation of primary damage and insufficient compensatory mechanisms, exhausting the organisms’ regenerative abilities (Guevara-Aguirre et al. 2011). Inflammation and altered mechanical properties of the cellular structure are two newly proposed hallmarks. They are derived from alterations in intercellular communication and can therefore be classified as integrative hallmarks. Similarly, autophagy is responsible for regulating various hallmarks and hence is considered within the same category (Gems and Magalhães 2021).

Considering the strong link between inflammatory processes (Potempa et al. 2017; Cheng et al. 2017) and dental pathologies resulting in difficulties eating food and functionality in advanced age (Sekundo et al. 2020b; Beker et al. 2019; Albani et al. 2021), oral health is important in the complex web of interactions.(see Fig. 3) It is vital in order to meet the nutritional requirements to sustain a healthy body (Sekundo et al. 2020b). For example, protein appears to be a key nutrient in maintaining muscle mass and reducing frailty (Hakeem et al. 2019; Tôrres et al. 2015; Wolfe 2012). Poor masticatory function leads to a reduction in protein intake, which could have a significant effect on morbidity and mortality (Wolfe et al. 2008; Coelho-Júnior et al. 2020; Motokawa et al. 2021).

Furthermore, periodontal disease is common in the elderly (Petersen and Kwan 2010). The pathological process in periodontitis is characterized by inflammation. It is conceivable that an improved ability to deal with low grade inflammation also results in lower levels of periodontal disease. The periodontal environment offers a unique portal of interaction between the immune system and bacterial flora. Oral health or a low grade of disease could be a potential surrogate for the bodies capabilities to deal with inflammatory processes in general. It is unclear at this point whether periodontitis is purely a feature of ageing.

An inflammatory state is central to many non-communicable diseases and may be the common denominator in these pathologies (Okamoto et al. 2019).

Attaining extreme longevity could be the result of an extraordinary capacity to deal with inflammation, preventing the establishment of systemic inflammation until very late in life.

**Fig. 4:**
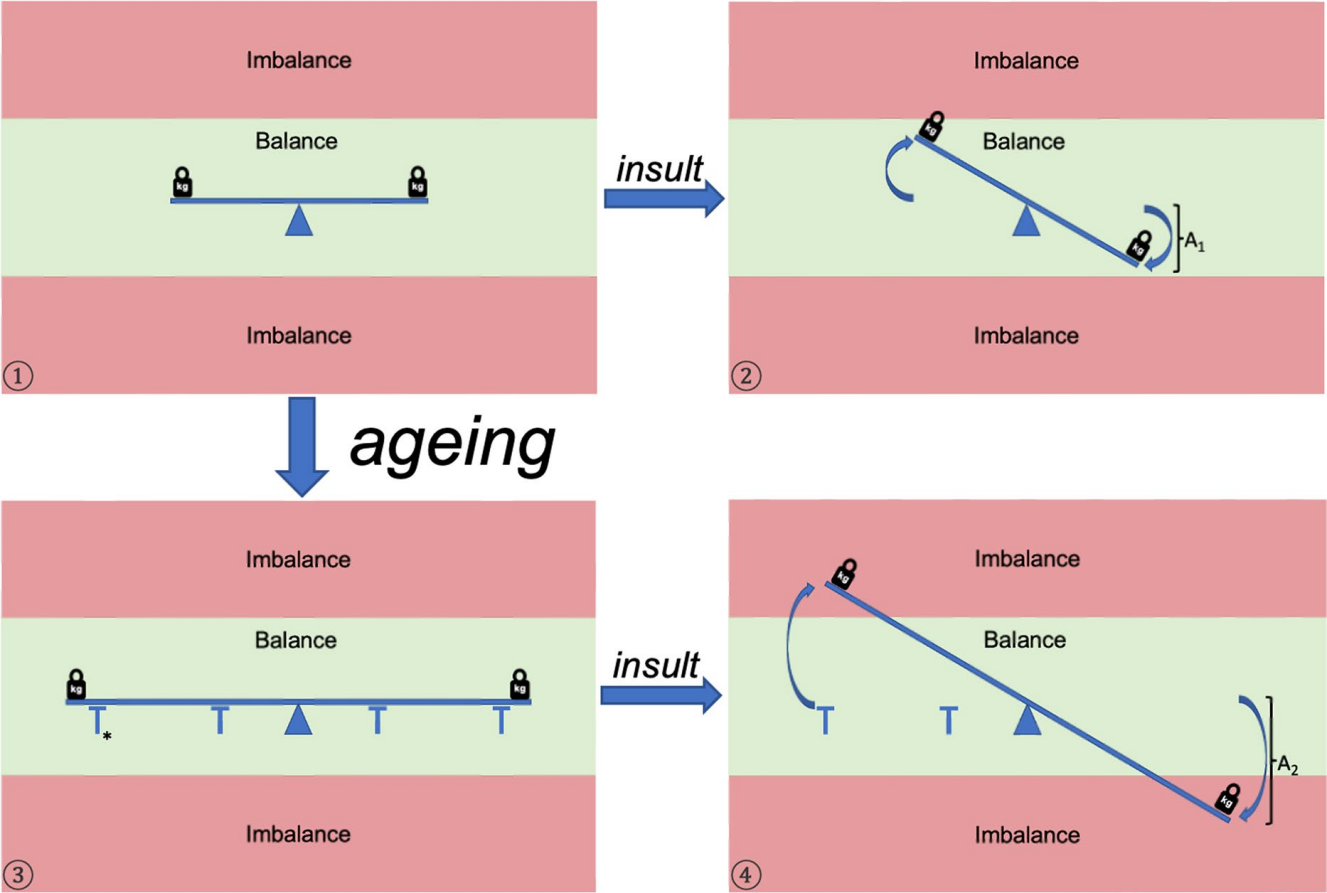
**Ageing: balance and imbalance**. The human body seeks a homeostatic state. **1)** Stressors (symbolized by weights) influence the biological state. Normally the organism has the capability to maintain balance. **2)** Insults e.g., disease, injuries, famine, etc. force the system out of its equilibrium. The impact of the stressing event has been symbolized by the amplitude A_1_. In the young resilient body, a conservation of a relative state of balance is often possible, offering the opportunity to regain an equilibrium e.g., fight disease, heal injuries, refeeding. **3)** Ageing increases the length of the lever, thus reducing resilience and leaving the organism more susceptible to insults. *Genetics, good dietary habits, exercise, purposeful living and social support can act as pillars of support, counteracting the increased vulnerability and enhancing the organisms´ compensatory capacity. **4)** Due to the increased susceptibility, the same insult results in a much larger negative impact (amplitude A_2_ > A_1_). This leads to a state of imbalance, exceeding the capacity of compensatory mechanisms. This results in incomplete recovery, chronic disease, frailty or death

Ageing is a dynamic process with multiple factors influencing a system striving for balance. (Fig. 4) The human body aims to re-establish homeostasis when unbalanced by stressors.

The older an individual becomes, the more difficult it is for the body to deal with insults, hence the greater the impact on the organism.

Certain behavioral patterns, like regular exercise, diet or purposeful living act as support to give the system more resilience. They act as pillars supporting the system. Retaining good oral health may be a supporting pillar enabling successful ageing.

## Implications for future research

As the communication between medical and dental research develops, connections between the fields will increase and ultimately contribute to a better understanding of the human body. Within centenarian research only a handful of studies take oral health into consideration (Micheelis 2011; Frese et al. 2020; Sekundo et al. 2020a; Nomura et al. 2020; Fabbri and Rabe 2007; Kaufman et al. 2014). The relationship between oral health and its potential influence on other modifying factors of ageing remains understudied.

Oral health could be an aggravating/complicating factor unbalancing the system. The masticatory system stands at the crossroads between nutrient uptake, inflammation and metabolic processes. Factors like IL-1beta are linked to periodontitis, systemic inflammation and immunosenescence (Potempa et al. 2017; Cheng et al. 2017; Kaur et al. 2013; Mirrielees et al. 2010). The role of oral health within this complex interaction needs to be investigated in centenarians.

There is a bi-directional interaction between systemic and oral inflammation. Inflammation acts as a potential accelerator, surrogate and end stage in the ageing process and therefore offers great potential for further exploration. Possible routes to consider are the implications of the molecular mechanisms of ageing in relation to periodontitis. Research has already begun to explore some of these avenues.

Links have been established between genomic instability and periodontal disease (Qiu et al. 2000; Xu et al. 2021). Epigenetic alterations and their role in periodontal disease are a focus in recent publications (Geng et al. 2020; Borba et al. 2019; Zhang et al. 2021; Jiang et al. 2022; Wang et al. 2021b). The implications of deregulated nutrient sensing and cellular senescence have also been explored (Coêlho et al. 2020; Azevedo et al. 2020; Zheng et al. 2019; Yang et al. 2021; Chen et al. 2021).

Another interesting avenue to examine the links between periodontitis and the molecular mechanisms of ageing is the aged mouse model. Research by Liang et al. suggests that aged mice offer a sufficient and low-cost model to research chronic periodontitis, (Kuang et al. 2020) whilst offering the opportunity to study mechanistical influences and interventions (Kuang et al. 2020; Zayed et al. 2020). Remarkable findings using this model demonstrated that a short-term inhibition of mTORC1 through rapamycin could reverse periodontal inflammation, periodontal bone loss and pathogenic microbial alterations (Zayed et al. 2020). Most aged mice models aim for a survival rate of 85–90%, (Liang 2010; An et al. 2020) providing the use-case in geriatric dentistry. However, this doesn´t necessarily represent the very exclusive cohort of centenarians, highlighting the need for focused dental research in this unique cohort.

Centenarians as models of successful ageing display low levels of inflammation over their lifespan. This suggests that they are better able to keep their system balanced despite insults. Considering the lack of data regarding probable differences in inflammatory markers, healthy centenarians are an intriguing cohort to study the relationship of inflammation and periodontal disease. Considering the role inflammation plays in the ageing process, investigating the prerequisite processes (hallmarks of ageing) could provide a better understanding of the relationship between oral disease and ageing.

Centenarians are a very exclusive cohort and the main challenge lies in the recruitment process. In previous studies the recruitment rate ranged between 13 and 97% (Frese et al. 2020; Nomura et al. 2020; Ono et al. 2021; Flurkey and J M., and Harrison, D E, 2007; Arai et al. 2010; Feng et al. 2022; Rong et al. 2019; Hartvigsen and Christensen 1976; Frisoni 2001). Notably, sampling method, recruitment strategy, invasiveness/effort of examinations and cultural background, differed between the studies, potentially accounting for the wide range of successful recruitment. Therefore, the study design should provide a minimal threshold, ideally visitations at home with a maximum time demand of 2–3 h. Considering the challenges of the recruitment process, it seems prudent to try to address as many scientifical questions as possible. This could be achieved by combing different diagnostical tools e.g., dental examination and measurement of inflammatory molecules in the blood during the same visitation.

## Conclusion

This review illustrates that oral health plays a significant role in reaching exceptional longevity, especially considering its influence on nutrient uptake (Wolfe et al. 2008; Coelho-Júnior et al. 2020; Motokawa et al. 2021). Ageing research has greatly advanced following the landmark paper describing the hallmarks of ageing by establishing a common language and concept of the ageing process (Guevara-Aguirre et al. 2011; Gems and Magalhães 2021) Aspects of the ageing process have been examined in centenarians, most notably the impact of chronic inflammation (Arai et al. 2015). Inflammation plays a key role in the pathogenesis of periodontal disease and its’ relation to other systemic comorbidities, (Zanni et al. 2003; Gondo et al. 2006) however the link between oral health and successful ageing remains underexplored. More research is needed to get a deeper understanding of the relationship between oral health and other processes known to play an important role in ageing. Translating and synthesizing this knowledge will be a challenge, but must include dental research in order to see the full picture. Similar to the Greek on the rosetta stone, which acted as the key in deciphering the hieroglyphs, oral health could be a translational piece to understand the process of ageing.

## Data Availability

The datasets used and/or analyzed during the current study are available from the corresponding author on reasonable request.

## Abbreviations

ADLs: Activities of daily living
AMP: Adenosine monophosphate
AMPK: 5’AMP-activated protein kinase
ATP: Adenosine triphosphate
BMI: Body mass index
CD: Cluster of differentiation
CMV: Cytomegalovirus
DNA: Desoxyribonucleic acid
FDI: Fédération Dentaire Internationale
GH: Growth hormone
HbA1c: Hemoglobin A1c
IGF1: Insulin-like growth factor 1
IL: Interleukine
INK4: Inhibitors of CDK4
IRP: Immune risk phenotype
LPS: Lipopolysaccharides
mRNA: Messenger ribonucleic acid
mTOR: Mammalian target of rapamycin
mTORC1: Mammalian target of rapamycin complex 1
NAD: Nicotinamide adenine dinucleotide
NCDs: Non-communicable diseases
NECS: New England Centenarian study
NK cell: Natural killer cell
RNA: Ribonucleic acid
ROS: Reactive oxygen species
SIRT: Sirtuin
SD: Standard deviation
T2DM: Type 2 Diabetes mellitus
TOOTH: The Tokyo Oldest Old Survey on Total Health
